# Influence of deposition rate on the structural properties of plasma-enhanced CVD epitaxial silicon

**DOI:** 10.1038/srep43968

**Published:** 2017-03-06

**Authors:** Wanghua Chen, Romain Cariou, Gwenaëlle Hamon, Ronan Léal, Jean-Luc Maurice, Pere Roca i Cabarrocas

**Affiliations:** 1 LPICM, CNRS, Ecole Polytechnique, Université Paris-Saclay, 91128 Palaiseau, France; 2 Total SA, Tour Michelet, 24 Cours Michelet – La Défense 10, 92069 Paris La Défense Cedex, France; 3 IPVF (Institut Photovoltaïque d’Ile-de-France), 92160 Antony, France

## Abstract

Solar cells based on epitaxial silicon layers as the absorber attract increasing attention because of the potential cost reduction. In this work, we studied the influence of the deposition rate on the structural properties of epitaxial silicon layers produced by plasma-enhanced chemical vapor deposition (epi-PECVD) using silane as a precursor and hydrogen as a carrier gas. We found that the crystalline quality of epi-PECVD layers depends on their thickness and deposition rate. Moreover, increasing the deposition rate may lead to epitaxy breakdown. In that case, we observe the formation of embedded amorphous silicon cones in the epi-PECVD layer. To explain this phenomenon, we develop a model based on the coupling of hydrogen and built-in strain. By optimizing the deposition conditions to avoid epitaxy breakdown, including substrate temperatures and plasma potential, we have been able to synthesize epi-PECVD layers up to a deposition rate of 8.3 Å/s. In such case, we found that the incorporation of hydrogen in the hydrogenated crystalline silicon can reach 4 at. % at a substrate temperature of 350 °C.

Epitaxial growth of high quality crystalline semiconductor layers has been developed in view of their applications in various domains such as telecommunications^1^ and photovoltaics^2^,^3^. Different epitaxial techniques have been developed, including liquid phase epitaxy (LPE)^4^, metal organic vapor phase epitaxy (MOVPE)^5^, hot-wire chemical vapor deposition (HWCVD)^6^, and molecular beam epitaxy (MBE)^7^. As far as epitaxy for photovoltaics is considered, two CVD-based techniques are widely studied, including atmospheric pressure CVD (APCVD) at temperatures around 1050 °C^8^ and radio-frequency (RF) plasma-enhanced CVD (PECVD)^9^, where the epitaxial growth at the low temperature of 200~400 °C is realized. The low-temperature epitaxy by PECVD (epi-PECVD) gains more and more interest recently as the epitaxy layers can be used as the optical absorber^2^,^3^,^10^,^11^,^12^ or the emitter^13^,^14^. There are three potential benefits associated to the low temperature process: low cost, reduced thermal expansion^12^, and sharp interface (heterostructure or doping profile) without inter-diffusion. Although, the low substrate temperature allows the growth of epi-PECVD with low thermal budget, it also brings a prominent limitation to the growth rate, which will limit the production capacity of the system in particular for thick absorber layers.

Indeed, in a PECVD process, an additional energy is provided in order to compensate the low thermal energy owing to low substrate temperature. As far as the deposition rate of epi-PECVD is concerned, several experimental parameters including substrate temperature, process pressure, inter-electrode distance, RF plasma power and gas flow rates have to be taken into account. Depending on the process conditions, different species including radicals, hydrogen, ions, and nanoparticles will react with the substrate in different amounts. Among these parameters, a critical one is the ion bombardment energy (IBE), which is proportional to the plasma potential (*V*_*pl*_). For Si ion-beam epitaxy, Rabalais *et al*. reported that there is a threshold of IBE for the defective low temperature epitaxy. This threshold will increase with the substrate temperature (*T*_*sub*_), for example, the upper limit of IBE increases from 20 to 30 eV when the substrate temperature is raised from 150 to 250 °C^15^. In the case of low temperature epi-PECVD using H_2_/SiH_4_ gas mixtures at 0.85 Torr, Bruneau *et al*. showed that the critical IBE is 30 eV for a substrate temperature of 175 °C, above which the epitaxy breakdown occurs^16^. Therefore, in this work, we address mainly two parameters (*V*_*pl*_ and *T*_*sub*_) in order to achieve high deposition rate of epi-PECVD at low temperature while avoiding epitaxy breakdown. The morphology and crystalline quality of epi-PECVD layers produced at three deposition rates were characterized by Scanning electron microscopy (SEM), high resolution transmission electron microscopy (HRTEM), Raman spectroscopy, as well as secondary ion mass spectrometry (SIMS). The evolutions of hydrogen concentration as well as the microstructure of the epi-PECVD layers with deposition rate in the range from 2 to 8.3 Å/s are studied.

## Results

In this work, three different deposition rates of epi-PECVD (low: 2 Å/s; medium: 4 Å/s and high: 8.3 Å/s) were studied. The detailed deposition parameters are listed in Table 1 of the methods section. *V*_*pl*_ is calculated to be 24 V; 45 V and 58 V by measuring the peak-to-peak voltage (*V*_*PP*_) and self-bias voltage (*V*_*DC*_) with the relation of *V*_*pl*_ = (*V*_*PP*_/2 + *V*_*DC*_)/2^17^.

### Low deposition rate (2 Å/s)

**Figure 1:**
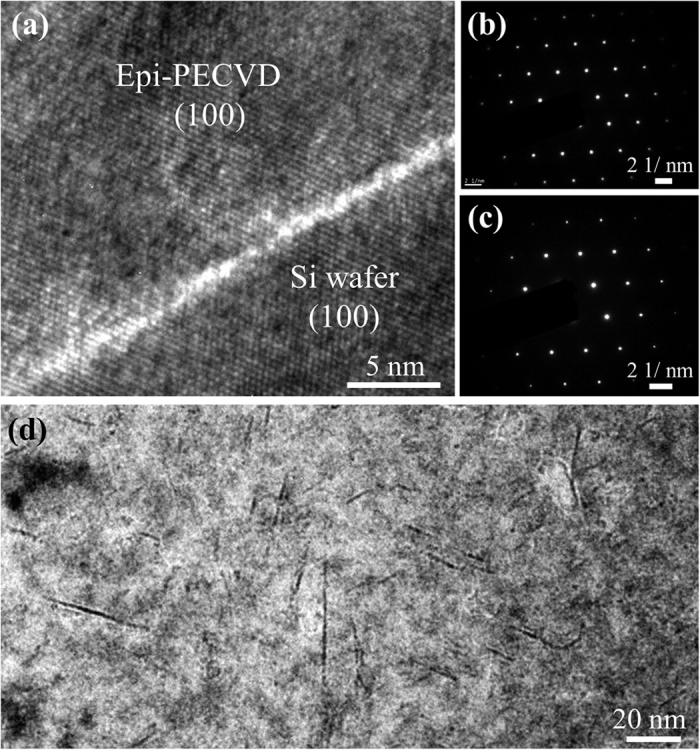
TEM images of epi-PECVD layer deposited on Si wafer at 2 Å/s. (**a**) High magnification TEM image showing the interface between c-Si wafer and epi-PECVD layer. (**b**) and (**c**) show the diffraction patterns of the epitaxial layer and the parent wafer, respectively. (*d*) Low magnification TEM image revealing hydrogen platelets in the bulk of the epi-PECVD layer.

Epi-PECVD layers were deposited at *V*_*pl*_ = 24 V and *T*_*sub*_ = 175 °C with a low deposition rate of 2 Å/s, which is our baseline process. To assess the crystallographic quality of epi-PECVD layers, HRTEM was used to characterize the as-deposited samples. It can be observed in Fig. 1(a) that epi-PECVD with the same crystallographic orientation as the substrate was deposited on a (100) c-Si wafer. However, we can also see clearly that the epi-PECVD/wafer interface was defective. We have reported recently the transfer of epi-PECVD layers onto low cost substrates (e.g. glass) via anodic bonding, by taking advantage this defective interface^3^. Figure 1(b) and (c) present the diffraction patterns of the epi-PECVD film and the c-Si wafer, respectively, where the sharp spots demonstrate the high crystalline quality of epi-PECVD. Figure 1(d) presents a low magnification TEM image showing the existence of hydrogen platelets in the bulk of the epi-PECVD layer. Note that a different observation condition is used by defocusing the TEM imaging for the purpose of enhancing the contrast of platelets.

**Figure 2:**
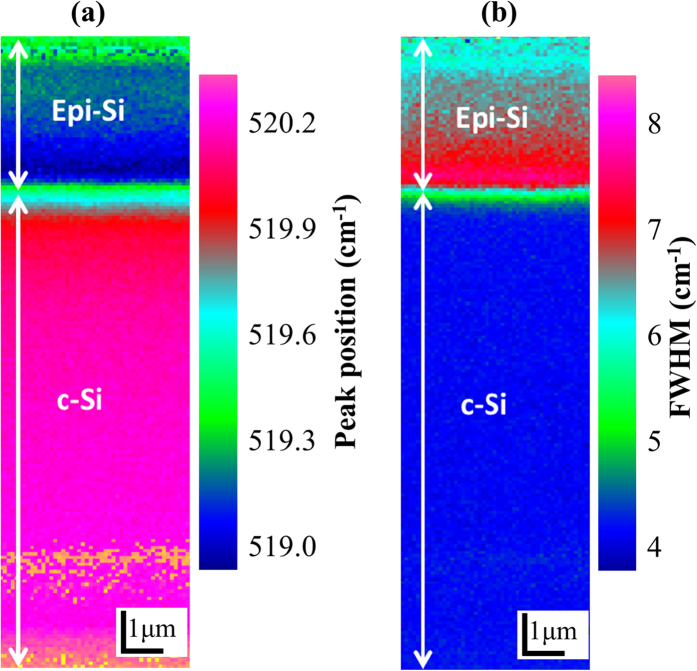
Cross-sectional Raman mapping of epi-PECVD layers. Raman mapping based on the c-Si Raman peak position (**a**) and its FWHM (**b**).

The crystalline quality of the layer obtained at low deposition rate was also characterized by two-dimensional Raman mapping across the interface as shown in Fig. 2(a) and (b), which maps the peak position and the full width at half maximum (FWHM) of the crystalline Si peak, respectively. The excitation wavelength was 532 nm. At λ = 532 nm, the absorption coefficient *α* equals to 9524 cm^−1^, where *α* is determined by fitting the spectroscopic ellipsometry data. Then, the light penetration depth (and thus probing depth) in epitaxial Si at λ = 532 nm can be calculated to be 1.05 μm by using *1/α*. The Raman mapping area is 21.5 × 5 μm^2^ with a pixel resolution of 0.2 × 0.2 μm^2^. The peak position (Fig. 2(a)) indicates a downshift at the interface and an upshift at the surface of epi-PECVD with respect to the c-Si. These shifts reveal that there is an evolution from a highly stressed interface to a less stressed epitaxial layer. The FWHM mapping (Fig. 2(b)) indicates a change of crystalline quality from the parent wafer to the surface of the epi-PECVD, starting from the wafer (4 cm^−1^) to the interface (~7.5 cm^−1^) until to the surface (~6.0 cm^−1^). Note that the wider peak relates to the presence of crystal defects. Therefore, Fig. 2(b) reveals that the crystalline quality of the epi-PECVD layer improves when moving away from the defective interface. The defective interface is due to the initial nucleation of hydrogenated c-Si on the parent wafer. Epi-PECVD with improving bulk crystalline quality with layer thickness, while keeping a defective interface, is beneficial as far as the transfer of thin c-Si solar cells is considered^3^.

### Medium deposition rate (4 Å/s)

**Figure 3:**
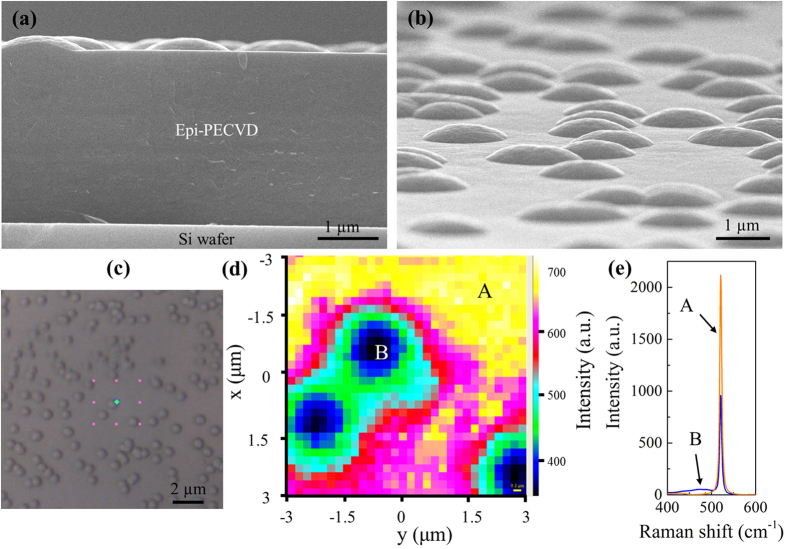
SEM image of epi-PECVD layer deposited at 4 Å/s. (**a**) The cross-section view and (**b**) the tilted view (15°). (**c**) Optical microscope image showing the Raman mapping area indicated by a pink-dotted-square. (**d**) Raman mapping of epi-EPCVD surface. (**e**) Raman spectra of region A (matrix) and region B (spherical cap).

Let us now study the epi-PECVD layer deposited at *V*_*pl*_ = 45 V and *T*_*sub*_ = 300 °C with medium deposition rate (4 Å/s). A cross-section SEM view is shown in Fig. 3(a) and a tilted view (15°) in Fig. 3(b), showing a very rough surface of epi-PECVD due to the existence of randomly distributed spherical caps. To characterize these spherical caps, Raman spectroscopy was used to map the epi-PECVD surface. We used the same Raman mapping parameters as in the previous section. Figure 3(c) represents the scanned epi-PECVD region, where the mapping area is indicated by a pink-dotted-square (top-viewing in optical microscope). The colors in Fig. 3(d) represent the intensity of the c-Si Raman peak at 521 cm^−1^. The spherical caps can be clearly distinguished by their lower peak intensity with respect to the matrix. The corresponding Raman spectra of region A (matrix) and region B (spherical cap) are shown in Fig. 3(e). The peak position and FWHM of region A are 521 cm^−1^ and 5.4 cm^−1^, respectively, which indicates that the matrix is crystalline. However, two peaks appear (at 520.9 cm^−1^ and 480 cm^−1^) on the spherical cap regions revealing that it corresponds to a mixture of two regions between a-Si and c-Si. Although, the Raman spectra allow us to distinguish the cap from the matrix, it can hardly tell us how the two regions of a-Si and c-Si are mixed. Moreover, the coupling of Raman and SEM gives no information whether the spherical cap is embedded in epi-PECVD or just covered on top of it. Therefore, we used TEM to characterize the cross-section the epi-PECVD layer.

**Figure 4:**
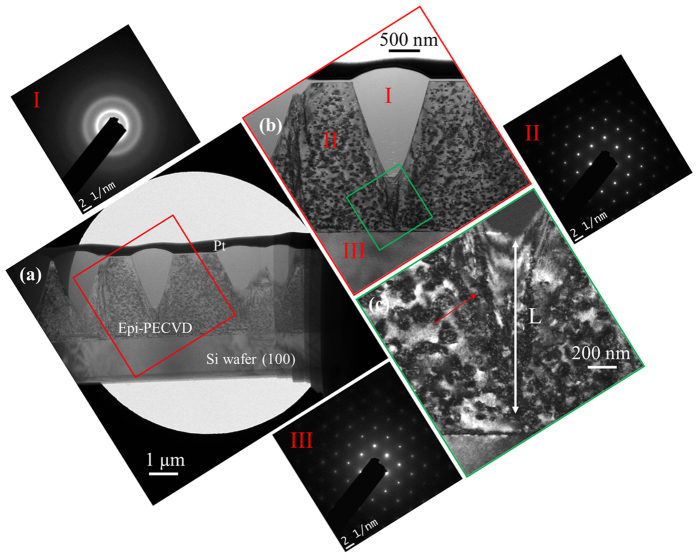
TEM characterization of epi-PECVD obtained at a deposition rate of 4 Å/s. (**a**) Cross-sectional TEM image showing the embed of amorphous cones in epi-PECVD layer. (**b**) Zoom on one single cone with diffraction patterns showing amorphous cone (I), (100) orientation for epi-PECVD (II) and c-Si wafer (III). Diffraction patterns of the amorphous cone (I), the epitaxial Si film (II) and the c-Si substrate (III) are also shown. (**c**) Enlarged view of the bottom of an amorphous cone showing the coalescence of hydrogen platelets (indicated with a red arrow). The measured hydrogen segregation length before breakdown is denoted as L.

Figure 4(a) shows the TEM image of the sample at low magnification, where different layers including the c-Si substrate, epi-PECVD and Pt layers can be observed. Interestingly enough, Fig. 4(a) shows that the previously observed spherical caps are the top part of the spherical cones. A zoom on a single cone is shown in Fig. 4(b). One may note there are two Pt protective layers deposited on the top of epi-PECVD. The first Pt layer (50 nm) is deposited via electron beam induced deposition (EBID), in order to avoid Ga ion implantation during the ion image acquisition, prior to the milling process. After that, a second Pt layer (500 nm) is deposited via ion beam induced deposition (IBID) at a higher deposition rate (higher bombardment energy). In Fig. 4(b), one can see plenty of dark zones due to the strained Si, which can be related to the excess of hydrogen atoms in the epitaxial layers. The diffraction patterns show that the epi-PECVD matrix has a (100) orientation, which is the same as that of the substrate, whereas the cone is amorphous. A zoom of the interface between epi-PECVD and wafer is shown in Fig. 4(c). We can see that the coalescence of hydrogen excess zones occurs at the bottom of the amorphous cone (guided by a red arrow in Fig. 4(c)).

Different breakdown mechanisms have been proposed in the literature, for example, isotropic deposition in low temperature CVD^18^, surface roughness in hydrogen free MBE^19^, strain in electron cyclotron resonance CVD^20^ and high ion energy in PECVD^16^. We consider that the epitaxy breakdown in low temperature PECVD is due to the coupling effect of hydrogen and built-in strain. The formation of cones can be explained as follows: 1) incorporation of hydrogen into epi-PECVD during deposition; 2) building of hydrogen-related strain; 3) precipitation of hydrogen leading to the formation of platelets; 4) coalescence of hydrogen platelets; 5) distortion of the Si lattice at same points on the surface of the growing sample due to the accumulated strain and 6) deposition of Si atoms on the disordered Si structure leading to the epitaxy breakdown.

**Figure 5:**
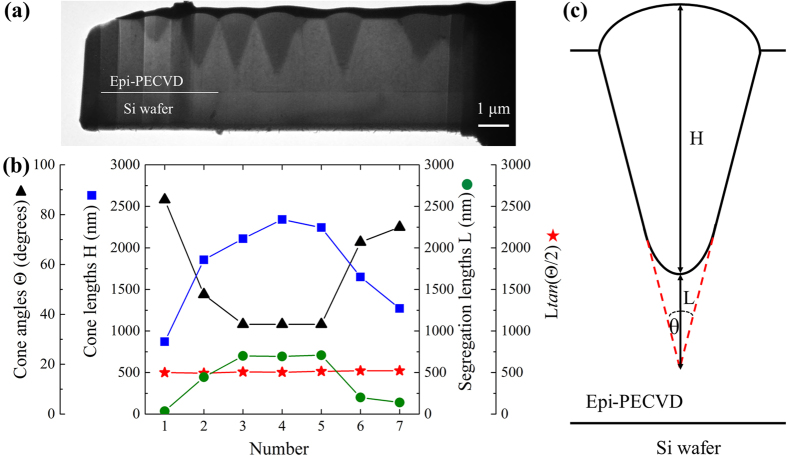
Distribution of amorphous cones in epi-PECVD layer. (**a**) TEM lamella shows the seven cones that have been measured. (**b**) The distribution of cone angles (triangles), cone heights (squares), segregation lengths (circles) and L*tan(θ/2*) of seven amorphous cones from (**a**). (**c**) Schematic illustration showing the measured parameters. The read dotted line indicates the hydrogen segregation region.

To study quantitatively the distribution of amorphous cones in epi-PECVD, we measured the cone height (*H*), cone angle (*θ*) and segregation length (L) of seven cones from the cross-sectional TEM (Fig. 5(a)). Figure 5(b) shows the values of the angle, height and length for these seven cones. The values of L*tan(θ/2*) are also shown. The schematic illustration of measured parameters is shown in Fig. 5(c). It can be seen that the cone angles are randomly distributed with a large distribution from 36° to 86°. This large cone angle distribution in our PECVD epitaxial break down is found to be completely different compared to the case of HWCVD, where all the cones had an angle of ~54° 18. Moreover, we observed that the cone height and segregation length are inversely proportional to the cone angle.

To express the distortion of the Si lattice (*Δl*), we can write an equation:


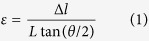


where *ε* is the built-in strain that we attribute to the segregation of hydrogen. Based on Hooke’s law, we can write:


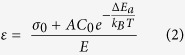


where σ_0_ is the stress when there is no incorporation of hydrogen and *E* is the elastic modulus of c-Si*. A* is a fitting coefficient. *C*_*0*_ is the hydrogen solubility in hydrogenated c-Si. *ΔE*_*a*_ is the segregation activation energy, *k*_*B*_ is Boltzmann’s constant and *T* is the deposition temperature. By combining Eqs 1 and 2, we have:


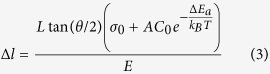


We considered that when *Δl* > *Δl*_*0*_, the break down occurs. At given temperature and hydrogen concentration, we have:


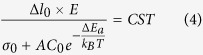


Therefore, we obtain *L*tan(*θ/2*) = *CST*, which is consistent with our experimental data (red stars) in Fig. 5.

### High deposition rate (8.3 Å/s)

**Figure 6:**
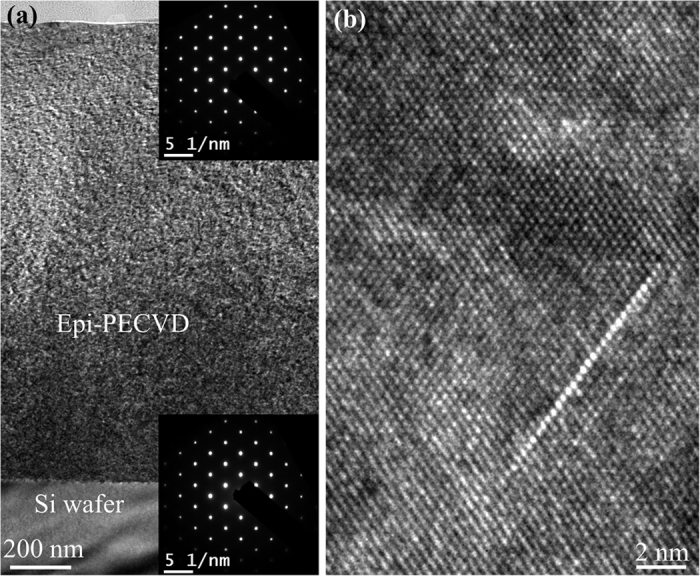
TEM characterization of epi-PECVD layer deposited at 8.3 Å/s. (**a**) Low magnification TEM image showing the high contrast between c-Si wafer and epi-PECVD layer. The insets show the TEM diffraction patterns of epi-PECVD layer and c-Si wafer respectively. (**b**) A zoom of epi-PECVD layer revealing that the bulk of the film has hydrogen platelets.

Now we move to the study of epi-PECVD deposited at *V*_*pl*_ = 58 V and *T*_*sub*_ = 350 °C at high deposition rate (8.3 Å/s). We also used TEM as the main characterization tool. A very high contrast between the epi-PECVD layer and the c-Si wafer is observed in the cross-sectional TEM shown in Fig. 6 (a). However, the diffraction patterns shown in the insets indicate that the epi-PECVD layer is monocrystalline with the same orientation (100) as the parent wafer. A zoom inside the epi-PECVD layer with atomic resolution in Fig. 6(b) shows the hydrogen platelets.

**Figure 7:**
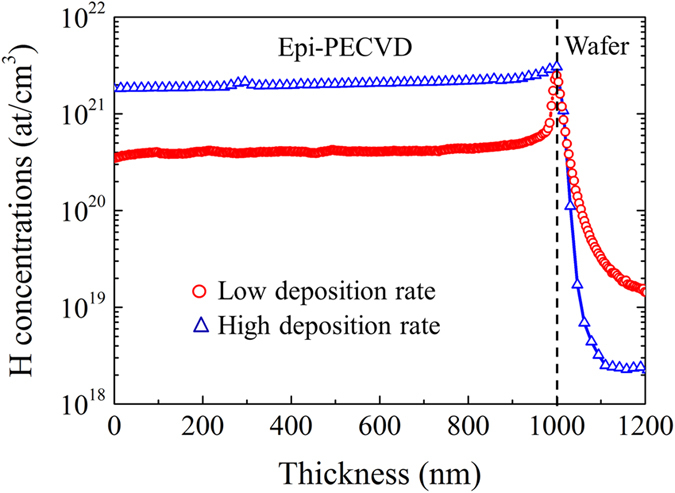
Concentration profiles of hydrogen atoms measured by SIMS of epi-PECVD layers. Two types of epi-PECVD layers deposited at low rate (2.0 Å/s) and high rate (8.3 Å/s) were measured.

Further evidence of hydrogen incorporation in epi-PECVD was obtained from SIMS, as shown in Fig. 7(a). We can see that the hydrogen concentration at the interface has the same value of 3 × 10^21^ at/cm^3^ for low and high deposition rate epi-PECVD layers. However, a big difference of hydrogen concentration inside the epi-PECVD layer is observed. We found that the hydrogen concentration inside epi-PECVD layer produced at high deposition rate is 2 × 10^21^ at/cm^3^ (4 at. % in c-Si:H) which is around six times higher than the one in epi-PECVD produced at low deposition rate of 3.5 × 10^20^ at/cm^3^ (0.7 at. % in c-Si:H). Note that the hydrogen concentration in c-Si:H can change its microstructure even for concentrations as low as 0.3 at. %^21^.

## Conclusion

In summary, we have synthesized epi-PECVD layers at three deposition rates: 2 Å/s; 4 Å/s and 8.3 Å/s by changing the substrate temperature and the plasma potential. For epi-PECVD layers deposited at 2 Å/s, we obtained a good crystalline quality which improved with thickness. However, for epi-PECVD layers deposited at 4 Å/s, we observed an epitaxy breakdown in the form of amorphous cones. We found that the cone angles are inversely proportional to the cone lengths. On the contrary, at 8.3 Å/s, the increase of growth temperature to 350 °C allowed to maintain the epitaxy growth. A model based on hydrogen and built-strain was developed to account for this epitaxy breakdown.

## Methods

### Material depositions

Prior to the deposition of epitaxial Si, the native oxide on the parent Si wafers (resistivity of 1–5 Ω∙cm, 280 μm thick, double side polished) was removed by dipping them into HF (5%) for 30 s. The wafers were then loaded inside a radio frequency (13.56 MHz) PECVD reactor. H_2_ and SiH_4_ are used as precursor and carrier gas, respectively. The deposition conditions for the low, medium and high deposition rate layers are listed in Table 1.

### Characterizations

SEM characterizations were performed using HITACHI S 4800 equipment. HRTEM characterizations were performed in a Jeol 2010 F (200 kV). The HRTEM samples were prepared by mechanical polishing followed by an ion milling process using a Precision Ion Polishing System (PIPS). In order to localize precisely the a-Si:H cones, the specimen of epi-PECVD with medium deposition rate was prepared by focused ion beam (FIB) equipped in the dual-beam SEM-FIB work station (NVISION 40 ZEISS SMT). The protective layers of Ga ions during FIB were deposited by Gas Injection System (GIS) by decomposing gaseous molecules (Me3)MeCpPt. Raman analysis (spectroscopy and mapping) were realized in HORIBA Scientific LabSpec 5.

## Additional Information

**How to cite this article**: Chen, W. *et al*. Influence of deposition rate on the structural properties of plasma-enhanced CVD epitaxial silicon. *Sci. Rep.* **7**, 43968; doi: 10.1038/srep43968 (2017).

**Publisher's note:** Springer Nature remains neutral with regard to jurisdictional claims in published maps and institutional affiliations.

## Figures and Tables

**Table 1:** PECVD deposition parameters for epi-PECVD layers.

Samples	Deposition rate (Å/s)	Substrate temperature (°C)	Pressure (Torr)	SiH_4_/(H_2_ + SiH_4_) ratio	Plasma potential (V)
Low	2	175	2.3	0.02	24
Medium	4	300	3.2	0.04	45
High	8.3	350	3.1	0.04	58
